# Screening and Procedural Guidance for Mitral Transcatheter Edge-to-Edge Repair (M-TEER)

**DOI:** 10.3390/jcm14144902

**Published:** 2025-07-10

**Authors:** Andromahi Zygouri, Prayuth Rasmeehirun, Guillaume L’Official, Konstantinos Papadopoulos, Ignatios Ikonomidis, Erwan Donal

**Affiliations:** 1 Cardiology Department, Université of Rennes, CHU Rennes, Inserm, LTSI–UMR 1099, F-35000 Rennes, France; andromaxizyg@gmail.com (A.Z.); prayuth.ras@nmu.ac.th (P.R.); guillaume.l.official@chu-rennes.fr (G.L.); 2 Echocardiography Laboratory, European Interbalkan Medical Center, 57001 Thessaloniki, Greece; papadocardio@gmail.com; 3 Echocardiography Laboratory, 2nd Cardiology Department, Medical School, National and Kapodistrian University of Athens, Attikon University Hospital, 12462 Athens, Greece; ignoik@gmail.com

**Keywords:** mitral regurgitation, TEER, transcatheter interventions, valvulopathies, structural heart diseases, transcatheter edge-to-edge repair, MitraClip

## Abstract

Mitral regurgitation (MR) is a common valvular heart disease associated with significant morbidity and mortality. For patients at high or prohibitive surgical risk, mitral transcatheter edge-to-edge repair (M-TEER) offers a less invasive alternative to surgery. This review outlines key aspects of patient selection and procedural planning for M-TEER, with a focus on clinical and echocardiographic criteria essential for success. Comprehensive imaging—especially 2D and 3D transesophageal echocardiography—is critical to assess leaflet anatomy, coaptation geometry, and mitral valve area. Selection criteria differ between primary and secondary MR and are guided by trials such as COAPT and MITRA-FR. Optimal outcomes rely on careful screening, anatomical suitability, and multidisciplinary evaluation. With growing experience and advancing technology, M-TEER has become a transformative option for treating severe MR in non-surgical candidates.

## 1. Introduction

Mitral regurgitation (MR) is a common heart valve disease and the second leading cause for cardiac surgery in Europe [1,2]. It is classified as either primary/degenerative (DMR) or secondary (SMR). DMR is caused by intrinsic structural abnormalities of the mitral valve apparatus—involving the leaflets and chordae tendineae—such as prolapse or flail (Carpentier type II), or restricted motion (type IIIa). SMR occurs in the presence of structurally normal leaflets and is the result of geometric or functional alterations of the left ventricle (LV) or the left atrium (LA). These alterations impair the closing forces or tether the mitral valve apparatus, leading to mitral regurgitation. The most common form is ventricular SMR (type IIIb), caused by left ventricular dysfunction and remodeling, leading to leaflet tethering. Atrial functional MR (AFMR), classified as type I, results from annular dilation due to atrial enlargement, often associated with atrial fibrillation or diastolic dysfunction. AFMR patients typically have a preserved ejection fraction and enlarged left atrium but normal left ventricular volume [3,4]. If left untreated, MR can cause left ventricular dysfunction, heart failure, and increased mortality [5,6,7].

Nearly 50% of the patients with MR cannot undergo surgery due to comorbidities, advanced age or high surgical risk. For these patients, transcatheter therapies offer a viable alternative, focusing on leaflet or annulus repair, or valve replacement. The most widely used technique is leaflet approximation (mitral transcatheter edge-to-edge repair/M-TEER) with more than 150,000 implantations worldwide. Two devices are currently available for commercial use, MitraClip (Abbott Vascular, Santa Clara, CA, USA) and the PASCAL (Edwards Lifesciences, Irvine, CA, USA), both targeting leaflet approximation through similar principles, but with distinct technical features. The purpose of this review is to discuss patient selection criteria for M-TEER and to outline the essential imaging and procedural guidance strategies necessary for successful intervention.

## 2. Echocardiographic Evaluation of MR

The echocardiographic evaluation of mitral regurgitation relies on an integrative approach combining qualitative, quantitative and semiquantitative parameters. The mechanism of MR can be identified through detailed qualitative assessment of mitral valve anatomy and subvalvular apparatus morphology [8].

**Figure 1:**
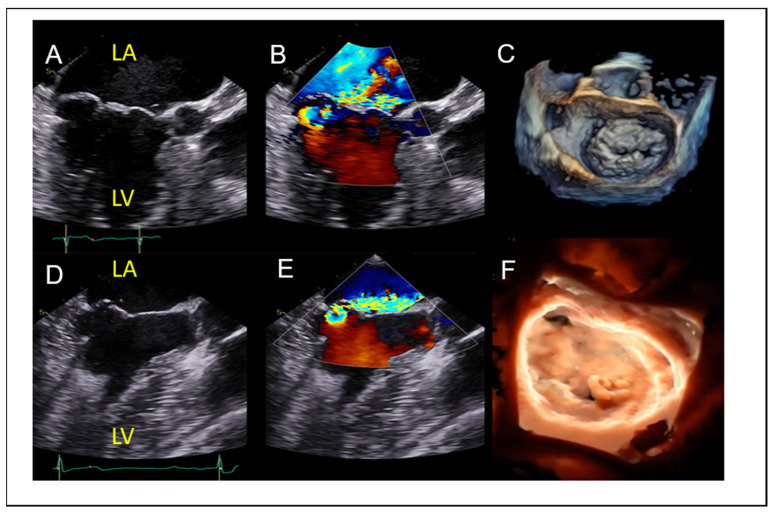
(**A**–**C**): Primary MR with multiscallop prolapse and diffusely thickened leaflets (Barlow disease). (**D**–**F**) Primary MR with P3 prolapse, thin leaflets and chordal rupture (fibroelastic deficiency). LA: left atrium, LV: left ventricle.

**Figure 2:**
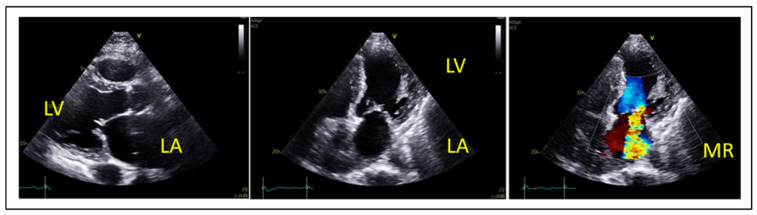
Severe secondary ventricular MR in a patient with ischemic cardiomyopathy due to akinesia of the inferolateral and lateral wall. LA: left atrium, LV: left ventricle, MR: mitral regurgitation.

The spectrum of degenerative (primary) mitral valve disease includes fibroelastic deficiency—characterized by thin leaflets and focal prolapse—to Barlow’s disease, which presents with diffusely thickened leaflets and prolapse involving multiple scallops, as illustrated in Figure 1. Another phenotype of DMR associated with malignant arrythmias is mitral annular disjunction (MAD) syndrome, which is characterized by bi-leaflet prolapse, systolic expansion and flattening of the mitral annulus, and a separation of at least 5 mm of the hinge point of the posterior mitral leaflet from the posterior wall of the left ventricle [9]. Other etiologies of degenerative MR include rheumatic valve disease, papillary muscle rupture in acute MR, leaflet perforation in the context of infective endocarditis, radiation- or drug-induced MR, congenital clefts, and connective tissue disorders. Secondary MR is defined by structurally normal mitral valve leaflets with impaired coaptation due to leaflet tethering or tenting, typically resulting from left ventricular or left atrial dilation (Figure 2). Eccentric regurgitant jets are commonly observed in primary MR (Figure 1), whereas central jets are more typical of secondary MR. However, in cases of ischemic secondary MR or posterior leaflet tethering, eccentric jets may also occur.

Semiquantitative parameters that support the diagnosis of severe MR include systolic flow reversal in pulmonary vein flow, dominant E-wave in mitral inflow (E-wave > 1.2 m/sec) and vena contracta width ≥7 mm (≥8 mm for biplane). Quantification of MR severity is mainly based on the PISA method, which is valid in central and round orifice jets [10]. Based on the PISA method, the effective regurgitant orifice area (EROA), the regurgitant volume (RVol) and the regurgitant fraction (RF) can be calculated [11,12,13].

Primary MR is defined as severe when EROA is ≥40 mm^2^, RVol ≥ 60 mL and RF ≥ 50%. For the definition of severe secondary MR, the same thresholds are proposed in ESC Guidelines 2021 and ACC/AHA guidelines for valvular heart disease [12,13]. Moreover, in ESC Guidelines 2021, lower thresholds (EROA is ≥30 mm^2^, RVol ≥ 45 mL) are proposed to be applied for defining severe secondary MR, in cases of elliptical regurgitant orifice area or low-flow conditions [12]. Additionally, increased EROA, RVol, RF are correlated with increased mortality in SMR patients [14,15]. An important risk-stratification algorithm based on the quantitative assessment of MR has been proposed by Bartko [15] and classifies SMR patients as low risk (EROA < 20 mm^2^, RVol < 30 mL), intermediate risk (EROA = 20–29 mm^2^, RVol = 30–44 mL) and high risk (EROA ≥ 30 mm^2^, RVol ≥ 45 mL). An RF < 50% defined low risk SMR patients, whereas an RF ≥ 50% defined high risk SMR patients [15].

**Figure 3:**
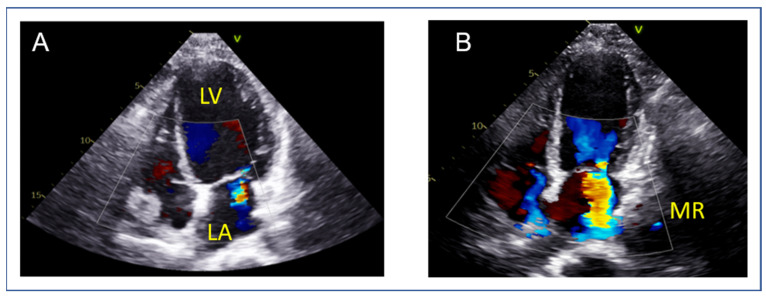
Ischemic dynamic MR unmasked by exercise echocardiography in a 66 y.o. patient with ischemic cardiomyopathy (LVEF = 25%) due to anterior infarct and exertional dyspnea NYHA III. (**A**) At rest, MR is mild, whereas (**B**) at 75 Watts the patient develops dyspnea upon aggravation of MR (EROA = 0.41 cm^2^, RVol = 39 mL) with significant elevation of pulmonary pressures (TR Vmax = 4.0 m/s, TR PeakPG = 65 mmHg, estimated SPAP = 70 mmHg). LA: left atrium, LV: left ventricle, MR: mitral regurgitation.

Another important aspect in the evaluation of SMR patients is the dynamic component of SMR [16,17] implying that different loading conditions (exercise, hypertension, volume overload), medical therapy and cardiac resynchronization therapy in cases of LV dyssynchrony, also have the potential to affect the severity of secondary MR. This dynamic component can be assessed by exercise echocardiography, especially in symptomatic SMR patients with less than severe MR at rest, as seen in the clinical example in Figure 3.

## 3. Indications for Intervention in Mitral Regurgitation

### 3.1. Indications for Intervention in Primary MR

Surgical mitral valve repair is the preferred treatment for severe primary MR. Indications for surgery in severe primary MR (VC ≥ 7 mm, RVol ≥ 60 mL, EROA ≥ 0.40 cm^2^, RF ≥ 50%) are driven by symptoms, or in asymptomatic patients by LV dysfunction (LVESD ≥ 40 mm or LVEF ≤ 60%), significant LA dilatation (LAVI > 60 mL/m^2^ or diameter > 55 mm) and pulmonary hypertension at rest (SPAP > 50 mmHg) when performed in a Heart Valve Center, and a durable repair is considered likely [12,13]. In MAD cases, surgical repair has the same results in terms of reducing the MR and thus it is indicated to treat these patients accordingly even though malignant arrythmias may persist [18]. However, a significant proportion of patients—particularly those of advanced age or with multiple comorbidities—are considered high-risk candidates for surgery. This has led to the emergence of transcatheter mitral valve interventions, among which transcatheter edge-to-edge repair (M-TEER) has become the most widely adopted percutaneous treatment strategy. In patients with severe primary mitral regurgitation (MR) who are symptomatic and considered inoperable or at high surgical risk, transcatheter edge-to-edge repair (M-TEER) is a recommended therapeutic option. According to current guidelines, this approach carries a Class IIa recommendation from the American College of Cardiology (ACC) and a Class IIb indication from the European Society of Cardiology (ESC) for those meeting appropriate anatomical criteria, as determined by the heart team [12,13].

### 3.2. Indications for Intervention in Secondary MR

In patients with severe secondary mitral regurgitation (SMR) undergoing coronary artery bypass grafting (CABG) or other cardiac surgery, concomitant mitral valve intervention is recommended (Class I, Level B, ESC guidelines) [12]. However, earlier trials have demonstrated that surgical repair of SMR is associated with high rates of reoperation, prompting many surgeons to favor valve replacement over repair in selected patients [19]. The recently announced MATTERHORN trial [20] compared M-TEER with surgical valve replacement in patients at acceptable surgical risk. It found that M-TEER provided comparable outcomes with fewer complications, supporting a growing trend toward transcatheter intervention as a first-line approach for SMR, even in operable patients.

For patients unsuitable for surgery but requiring revascularization, the heart team may consider percutaneous coronary intervention (PCI) and/or transcatheter aortic valve implantation (TAVI), followed by M-TEER, depending on the patient’s clinical profile (Class IIa indication, ESC guidelines). M-TEER is also recommended for selected heart failure (HF) patients who remain symptomatic despite guideline-directed medical therapy (GDMT), including cardiac resynchronization therapy (CRT). With regard to atrial functional mitral regurgitation (AFMR), this entity is not distinctly addressed in current guidelines, and no formal consensus exists to support a surgical or transcatheter approach. However, several prior studies [4,21,22] have demonstrated procedural feasibility and success of M-TEER in AFMR patients. At present, therapeutic decisions in this subgroup remain at the discretion of the multidisciplinary heart team.

M-TEER offers a minimally invasive alternative to surgery, with evidence from key trials such as COAPT and RESHAPE HF2 showing significant improvement in symptoms, reduced hospitalizations, and lower all-cause mortality when compared with medical therapy alone [23,24]. Landmark studies like COAPT and MITRA-FR have defined the role of M-TEER in patients with HF and persistent SMR, despite optimal GDMT or CRT [23,25].

According to the latest ESC and ACC/AHA guidelines, M-TEER is recommended (Class IIa) in symptomatic HF patients with severe SMR, favorable anatomical features, and evidence of responsiveness to M-TEER—criteria established in the COAPT trial [12,13,23].

## 4. Patient Selection and Screening Criteria

Importantly, optimal M-TEER outcomes depend on careful patient selection and preprocedural echocardiographic screening. A multidisciplinary heart team is essential for patients’ evaluation and should include cardiac imaging specialists, interventional cardiologists, cardiac surgeons, HF specialists and electrophysiology specialists as well.

### 4.1. Selection in Primary Mitral Regurgitation (PMR)

Primary MR results from intrinsic abnormalities of the mitral valve leaflets, including prolapse, flail, perforation, or restriction, classified using the Carpentier system. M-TEER is currently indicated for symptomatic severe PMR in patients at high or prohibitive surgical risk, as determined by clinical scores (STS, EuroSCORE II) and frailty indices [12,13]. In this population, M-TEER has shown consistent safety and effectiveness, particularly in improving symptoms and reducing MR severity, with subsequent reverse remodeling of the left ventricle [26].

The EVEREST II trial laid the groundwork for PMR treatment with M-TEER, demonstrating non-inferiority to surgery in selected patients with favorable anatomy—central jet origin, adequate leaflet length, and minimal calcification [27]. However, contemporary practice increasingly expands beyond these initial anatomical criteria due to advances in imaging, device design, and operator experience.

Procedural success remains the key determinant of long-term outcome. Therefore, careful anatomical assessment—including segment involvement, leaflet length, mobility, flail gap, and calcification—is essential. Ongoing improvements in TEER systems and accumulated procedural expertise have enabled treatment of anatomies once deemed unsuitable. Selection should ultimately be guided by a heart team, balancing anatomical feasibility with surgical risk and patient preference.

### 4.2. Selection in Secondary Mitral Regurgitation (SMR)

Clinical and echocardiographic predictors of favorable outcomes were first established by the COAPT trial, which focused specifically on ventricular secondary mitral regurgitation (V-SMR) [23]. This study enrolled heart failure patients with ischemic or non-ischemic cardiomyopathy and reduced left ventricular ejection fraction (LVEF) between 20–50%. Patients with LVEF < 20% were excluded due to the limited likelihood of therapeutic benefit. The left ventricle was required to not be excessively dilated, with an end-systolic diameter <70 mm, and mitral regurgitation had to be clinically significant—defined as moderate-to-severe (grade ≥ 3+) with an effective regurgitant orifice area (EROA) >30 mm^2^ and regurgitant volume (RVol) > 45 mL, or severe (grade 4+) with EROA > 40 mm^2^ and RVol > 60 mL. Eligible patients were symptomatic (NYHA Class II–IV, ambulatory), receiving guideline-directed medical therapy (GDMT) and cardiac resynchronization therapy (CRT) if indicated. Other inclusion criteria included at least one HF hospitalization in the previous year, elevated natriuretic peptides, and mitral valve anatomy suitable for M-TEER.

In light of COAPT and MITRA-FR trials, the concept of disproportionate and proportionate MR emerged to explain the contradictory results of these trials [28]. Disproportionate MR refers to a greater degree of MR than expected for the degree of LV dilatation and suggests a higher likelihood of benefit from M-TEER. In contrast, proportionate MR reflects MR severity that aligns with the degree of LV dilatation, suggesting that optimal medical therapy, rather than intervention, may be the preferred treatment approach [28].

Disproportionate MR can be quantified by using volumetric ratios, such as EROA/LVEDV or RVol/LVEDV. Namazi et al. [29] demonstrated that patients with a regurgitant volume to left ventricular end-diastolic volume (RVol/LVEDV) ratio greater than 20% had poorer survival under medical management. This finding suggests that disproportionate mitral regurgitation—defined by more severe regurgitation relative to the degree of LV dilatation—is associated with improved all-cause mortality outcomes when treated with mitral regurgitation-correcting interventions. Similarly, Berrill et al. found that disproportionate MR defined by EROA/LVEDV > 0.14 mm^2^/mL is associated with worse prognosis in patients with acute heart failure [30]. To guide clinical decision-making, COAPT-eligible vs. ineligible patient profiles (Table 1) should be systematically evaluated during screening. Nevertheless, exclusion from treatment should not be based solely on failure to meet COAPT criteria.

The RESHAPE-HF2 trial expanded the scope of M-TEER by including patients with symptomatic HF and moderate FMR (EROA 25 mm^2^) [31]. The results show a clear clinical benefit and reduction of hospitalizations and significant decline in the composite rate of hospitalizations and death [24,31]. These findings are consistent with the EXPAND study, which also demonstrated benefit in moderate MR patients treated with TEER, including evidence of reverse LV remodeling—a key therapeutic goal in HF management [32]. Similar reverse remodeling effects have been reported in smaller cohort studies [33,34], including strain imaging research by Papadopoulos et al. [35], suggesting TEER’s value even in patients with relatively preserved LV function without extensive fibrosis [36].

Importantly, in patients with severe secondary mitral regurgitation (SMR) and advanced heart failure who do not meet COAPT criteria, M-TEER may still be considered for symptomatic relief or as a bridge to heart transplant (BTT) or left ventricular assist device (LVAD) therapy, as per ESC guidelines (class IIb recommendation). The International MitraBridge Registry demonstrated the safety and feasibility of MitraClip in this population, showing that two-thirds of patients were free of adverse events at one year, and, notably, that 23.5% demonstrated sufficient clinical improvement to no longer require transplantation [37]. Another group of patients that could benefit is patients presenting with cardiogenic shock and moderate-to-severe mitral regurgitation. In a multicenter analysis, transcatheter mitral valve repair was associated with improved short- and long-term survival in this high-risk cohort [38]. However, further prospective, randomized trials are warranted to validate these findings and inform future guideline recommendations.

## 5. Anatomical Considerations for TEER

Before selecting patients with primary or secondary mitral regurgitation (MR) for transcatheter edge-to-edge repair (TEER), a thorough anatomical evaluation is essential to ensure procedural success [27,39]. Key echocardiographic parameters should be systematically assessed, including:

- Posterior leaflet length: A minimum of 7 mm (ideally > 10 mm) is typically required for adequate leaflet grasping.
- Flail gap and flail width: Severe primary MR may demonstrate a flail gap > 10 mm and width > 15 mm, which has traditionally limited eligibility but can be managed in experienced centers.
- Coaptation depth and coaptation length: Excessive coaptation depth (>11 mm) or reduced coaptation length (<2 mm) may pose procedural difficulty in secondary MR.
- Mitral valve area (MVA) and pressure gradient (PG): MVA < 4.0 cm^2^ may raise concern for post-procedural mitral stenosis, especially in patients requiring multiple devices. Cut-off values of 3.0 cm^2^ for MVA and 4 mmHg for mean PG are used to consider a patient ineligible for this method.
- Mitral annular and leaflet calcification: These may hinder adequate device deployment and leaflet grasping.
- Mitral annulus dimensions: Small dimensions of annulus (annulus area, anterior–posterior and medial-lateral diameters) should also be considered in the screening process.

Multiplanar reconstruction (MPR) and 3D transesophageal echocardiography (3DTOE) are indispensable tools in accurately measuring these parameters and ensuring favorable anatomy and procedural outcomes. Anatomical criteria are presented in Table 2.

## 6. Implications for Clinical Practice

Low-volume centers should begin with central jets, located at the A2/P2 segments, with mitral valve area (MVA) above 4 cm^2^, with posterior leaflet length above 10 mm and with no calcification in the grasping zone [40]. For degenerative MR, flail gap should be below 10 mm and for secondary MR the tenting height should not exceed 10 mm for beginner cases. Commissural jets (A1/P1, A3/P3) represent more challenging anatomies, as well as MVA 3.0–4.0 cm^2^, posterior leaflet length 7–10 mm or cleft and presence of annulus calcification. High-volume centers and experienced teams should treat complex cases, such as patients with MVA < 3.0 cm^2^, posterior leaflet length < 7 mm and cleft, calcification in the grasping zone, Carpentier IIIb, and Barlow disease with multiple segments prolapsing, after a case-by-case analysis by an experienced heart team [40]. However, there are some limitations of the method even for experienced centers: red, or unsuitable, cases might need transcatheter annuloplasty or even transcatheter MV replacement. Finally, it should be mentioned that, according to ESC and AHA guidelines [12,13], these implications concern only patients with primary MR that are at high surgical risk. If the patient is considered suitable for surgery, then the current indicated treatment is surgical repair or replacement of the valve.

## 7. Preprocedural Echocardiographic Assessment

Comprehensive echocardiographic assessment is the cornerstone of transcatheter edge-to-edge repair (TEER) planning. In both primary/degenerative mitral regurgitation (DMR) and secondary mitral regurgitation (SMR), accurate anatomical characterization informs feasibility and procedural strategy. Preprocedural imaging should systematically evaluate leaflet anatomy, valve area, and coaptation geometry. This section focuses on essential 2D and 3D transesophageal echocardiography (TEE) views, with an emphasis on reproducible measurements relevant to patient selection and procedural success.

### 7.1. Mitral Valve Anatomy

**Figure 4:**
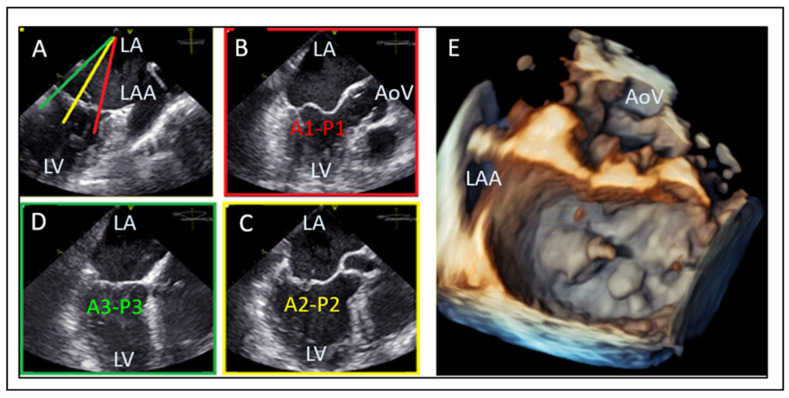
Segmental assessment of the mitral valve. (**A**) Mid-esophageal commissural view with biplane imaging allows systematic segmentation from the lateral commissure (noted by the left atrial appendage). (**B**–**D**) Biplane cuts demonstrate sequential leaflet scallops: A1–P1 (red line, (**B**)), A2–P2 (yellow line, (**C**)), and A3–P3 (green line, (**D**)). (**E**) Three-dimensional “en face” view of the mitral valve from the left atrial perspective provides a comprehensive anatomical context and spatial orientation of scallops. LA: left atrium, LV: left ventricle, LAA: left atrial appendage, AoV: aortic valve.

The mid-esophageal commissural TOE view allows for the precise segmentation of mitral valve anatomy and identification of different scallops. The use of biplane imaging and scanning from lateral to medial commissure allows for the localization of mitral valve pathology between different scallops of the anterior and posterior leaflet. Additionally, the 3D “en face” render view provides a “surgeon’s view” for intuitive localization of A1–P1, A2–P2, and A3–P3 scallops, as seen in Figure 4. For correct orientation into the dataset, two landmarks are used for biplane and 3D evaluation of MV: the aortic valve and the left atrial appendage (LAA). In biplane imaging, the lateral part of the valve is close to the left atrial appendage while the medial part is on the other side. The 3D “Surgeon’s” view requires an aortic valve position at 12 o’ clock and an LAA at the left side of the screen in an atrial perspective of the valve. This orientation allows one to distinguish lateral from medial, as well as anterior from posterior.

### 7.2. Posterior Leaflet Length

**Figure 5:**
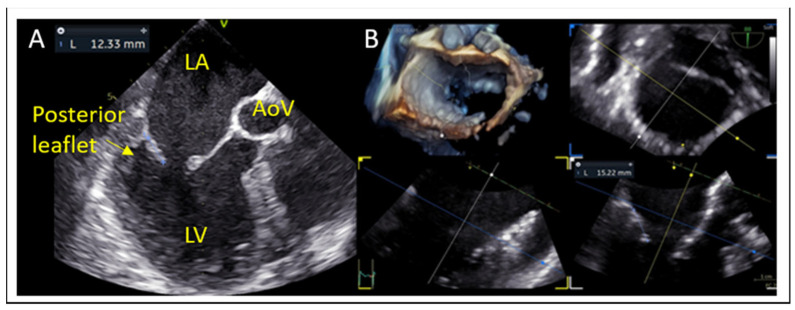
(**A**) Posterior leaflet length measurement using 2D and (**B**) multiplanar reconstruction (MPR) at grasping zone. LA: left atrium, LV: left ventricle, AoV: aortic valve.

Posterior leaflet length is measured from the annular hinge point to the free edge in mid-esophageal long-axis and this is corroborated on 3D multiplanar reconstruction (MPR) (Figure 5). Posterior leaflet length ≥ 10 mm is considered ideal for leaflet grasping [27].

### 7.3. Mitral Valve Area and Gradient

**Figure 6:**
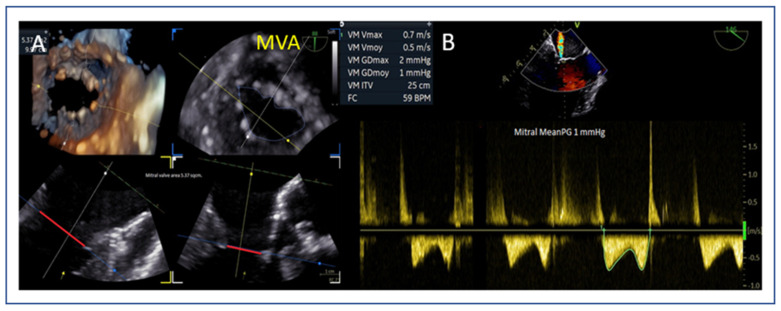
(**A**) Mitral valve area measurement using MPR. (**B**) Baseline mean mitral valve gradient using CW Doppler. MVA: mitral valve area.

Planimetric MVA should exceed 4.0 cm^2^ to avoid post-procedural mitral stenosis as TEER generally reduces the MVA by 50% (Figure 6A). The mean gradient should also be recorded at baseline (Figure 6B) with a cut-off value > 4 mmHg to be considered as a predictor for post-operative stenosis. A valve area between 3.0–4.0 cm^2^ is considered acceptable but requires caution, while <3.0 cm^2^ is considered as unsuitable for M-TEER [41].

### 7.4. Flail Gap and Width (PMR)

**Figure 7:**
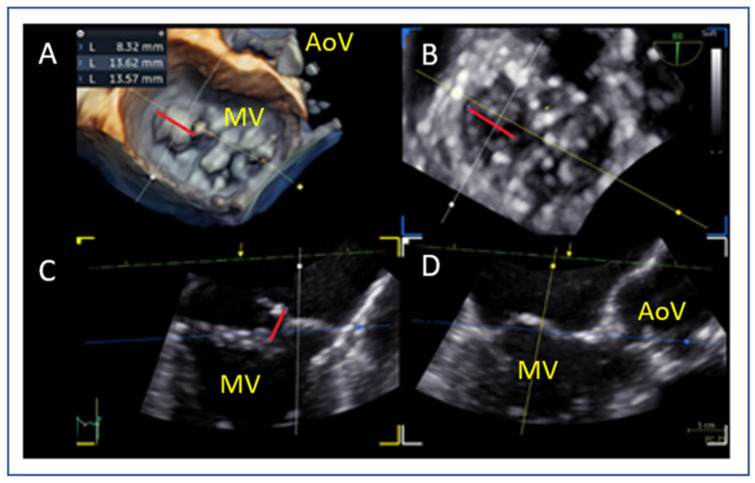
(**A**,**B**) Measurement of flail width in primary MR (red line). (**C**,**D**) Measurement of flail gap (red line). MV: mitral valve, AoV: aortic valve.

Flail width (Figure 7A,B) and flail gap (Figure 7C,D) in PMR are best assessed using 3D multiplanar reconstruction (MPR). Flail gap < 10 mm and width < 15 mm often allow for single clip implantation, while a larger width may require multiple devices [42].

### 7.5. Tenting Height and Coaptation Length (SMR)

**Figure 8:**
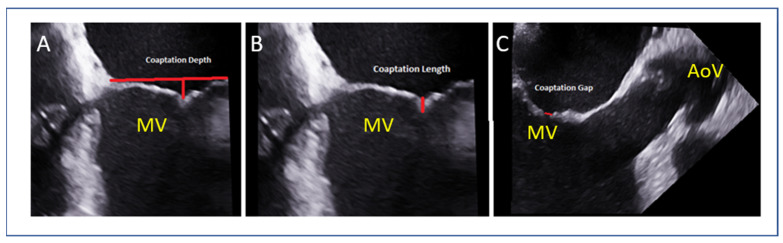
(**A**) Measurement of coaptation depth (or tenting height) in secondary MR (red line). (**B**) Measurement of coaptation length (red line). (**C**) Measurement of coaptation gap (red line). MV mitral valve, AoV: aortic valve.

Coaptation depth or tenting height, coaptation length and coaptation gap are additional parameters that have to be evaluated in secondary MR (Figure 8). These reflect leaflet tethering from LV remodeling. A tenting height < 10 mm and coaptation length > 2 mm predict better leaflet approximation by M-TEER.

## 8. Anatomical Challenges

- Posterior Leaflet Cleft-like Indentation

**Figure 9:**
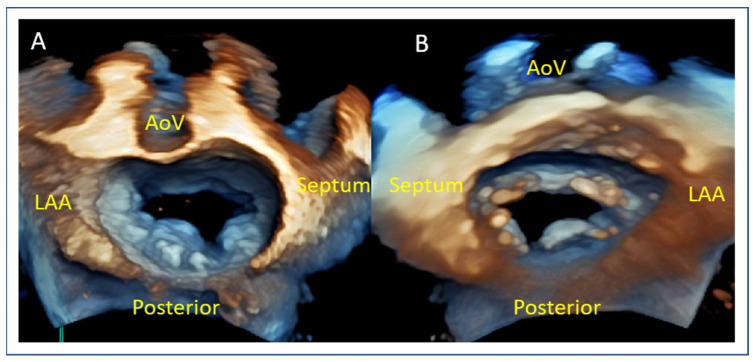
Three-dimensional “en face” mitral valve shows cleft-like indentation at the junction of the P2 and the P3 segment. (**A**) Atrial view or surgeon’s view and (**B**) ventricular view. LAA: left atrial appendage, AoV: aortic valve.

The posterior leaflet typically has two indentations that differentiate the scallops. A cleft-like indentation is defined as having a depth of at least 50% of the adjacent scallops [43,44] and 3D imaging is the best option to recognize such abnormalities (Figure 9). This feature makes grasping challenging and may lead to residual mitral regurgitation (MR).

- Leaflet and Annular Calcification

**Figure 10:**
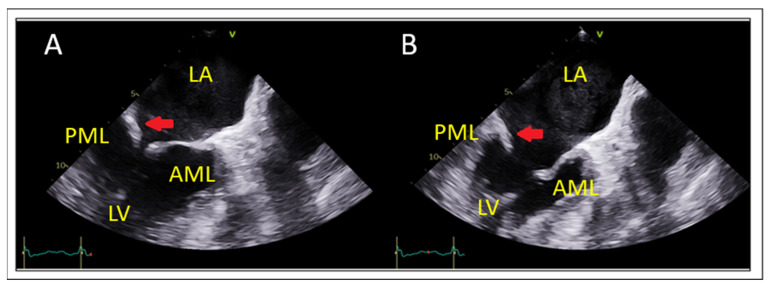
(**A**) Comparative assessment of posterior leaflet motion in (**A**) systolic and (**B**) diastolic phases. The posterior leaflet, indicated by a red arrow in both images, demonstrates a complete lack of movement throughout the cardiac cycle. LA: left atrium, LV: left ventricle, AML: anterior mitral leaflet, PML: posterior mitral leaflet.

Presence of calcium in the grasping zone or annulus (Figure 10) limits the device stability and increases both the risk of detachment and mitral stenosis [45].

- Adequate but Tethered Leaflets

**Figure 11:**
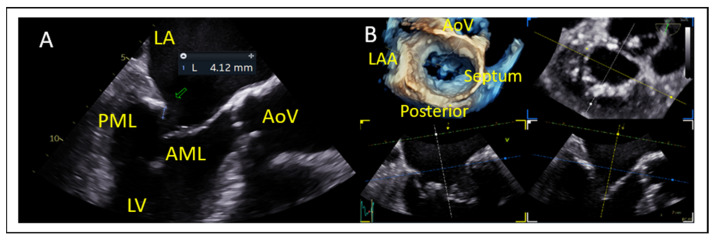
(**A**) The posterior annulus (green arrow) is calcified and extended into the posterior leaflet, resulting in a shorter effective posterior leaflet for grasping in 2D image and (**B**) in 3D with multiplanar reconstruction. LA: left atrium, LV: left ventricle, AML: anterior mitral leaflet, PML: posterior mitral leaflet, LAA: left atrial appendage, AoV: aortic valve.

Posterior leaflet length may be sufficient, but severe tethering reduces coaptation and grasping success (Figure 11).

## 9. Intraprocedural Guidance

### 9.1. Transseptal Puncture

**Figure 12:**
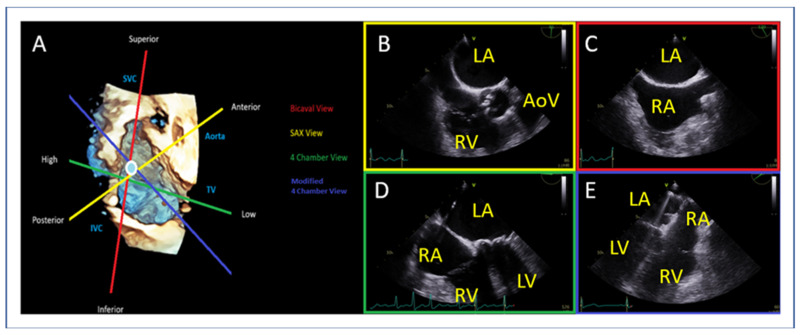
(**A**) The circle represents optimal transseptal puncture site (superior and posterior). (**B**) The short axis view guides anterior (towards the aorta) or posterior position, (**C**) the bi-caval view guides superior or inferior position, (**D**) the 4-chamber view is used for measurement of puncture height from the level of annulus and, (**E**) when the puncture site is positioned more superiorly than typically expected and the four-chamber view fails to adequately delineate both the puncture site and the annulus, a modified 135–150° view is employed to optimize alignment, thereby enabling precise visualization and measurement. LA: left atrium, LV: left ventricle, RA: right atrium, RV: right ventricle, AoV: aortic valve.

The transseptal puncture is an essential step in the M-TEER procedure (Figure 12). When undertaken correctly, this facilitates access and alignment for device delivery, streamlining the procedure. However, if not performed accurately, it can induce challenges that complicate the procedure. Precision at this stage is key to ensuring procedural success.

**Figure 13:**
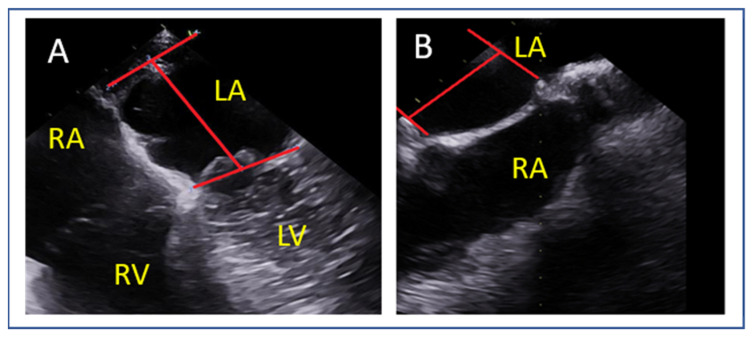
(**A**) Four-chamber view and (**B**) modified 135–150° view for measurement of the height of transeptal puncture from the level of annulus (vertical red line). LA: left atrium, LV: left ventricle, RA: right atrium, RV: right ventricle.

Different mitral valve pathologies and target segments necessitate specific transseptal puncture locations to optimize access and alignment. A superior and posterior puncture at approximately 4 cm serves as the default strategy [46] (Figure 12 and Figure 13).

**Figure 14:**
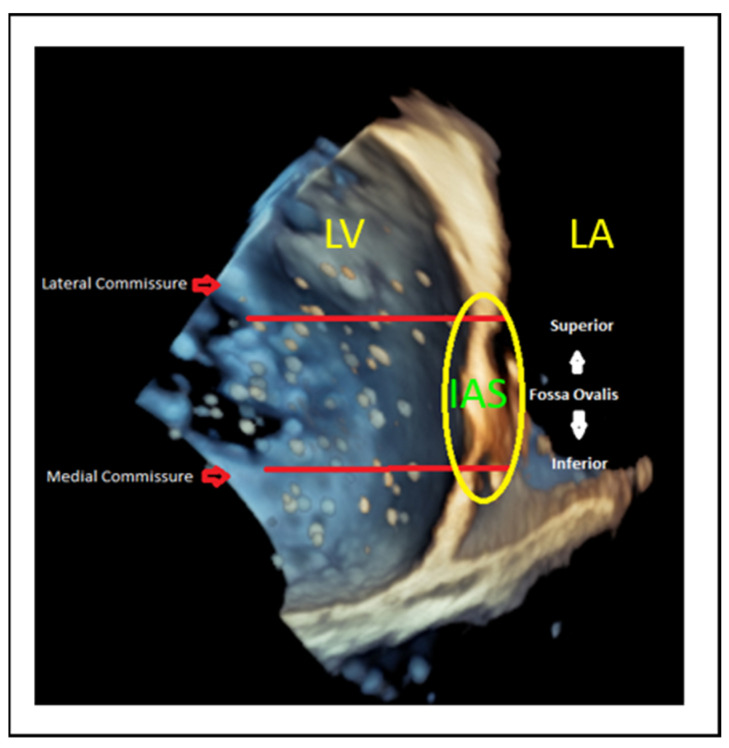
Superior puncture position offers better alignment to the lateral commissure, whereas inferior puncture position offers better alignment to the medial commissure. These adjustments are crucial to tailoring the procedure based on the targeted segment and ensuring optimal outcomes. LA: left atrium, LV: left ventricle, IAS: interatrial septum.

However, adjustments are required based on the targeted segment (Figure 14), as follows:

- Lateral commissure: A superior and lower puncture height, approximately 3.5 cm, is preferred to facilitate access [46].
- Medial commissure: A more inferior puncture, closer to the inferior vena cava (IVC), with a higher height of 4.5–5 cm is recommended for better alignment [47].
- Ventricular functional MR: The puncture height should be set 1 cm lower than the usual height to match the coaptation depth [46].

### 9.2. Navigating the Device

**Figure 15:**
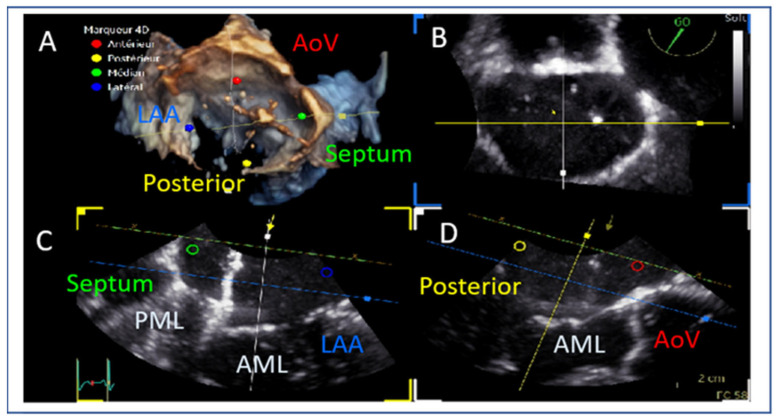
(**A**,**B**) Navigating the guiding catheter and the device towards the mitral valve. (**C**) Medial–lateral orientation (blue = lateral towards the left atrial appendage and green = medial towards the septum). (**D**) Anterior–posterior orientation (red = anterior towards the aortic valve and yellow = posterior). AoV: aortic valve, LAA: left atrial appendage, AML: anterior mitral leaflet, PML: posterior mitral leaflet.

Echocardiographic guidance of the device towards the mitral valve requires simultaneous use of commissural and long-axis TOE views in order to target the anterior–posterior and medial–lateral part of the valve. Biplane imaging or live 3D guidance with MPR can provide all of the necessary information for this step of the procedure (Figure 15). The device is advanced towards the mitral valve this way and this approach optimizes alignment and minimizes the risk of injury of neighboring structures.

### 9.3. Device Alignment and Implantation

**Figure 16:**
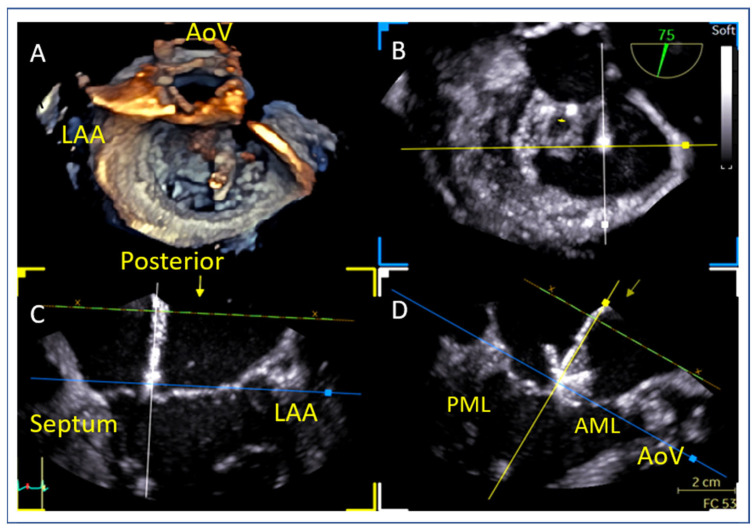
Multi-planar reconstruction (MPR) for device orientation and perpendicularity. (**A**) Three-dimensional en-face view of the mitral valve, demonstrating device orientation, (**B**) short axis projection of the orientation of the device, (**C**) Bi-commissural view for medial-lateral position as-sessment of the device, (**D**) Long-axis view for anterior-posterior position assessment of the device. AoV: aortic valve, LAA: left atrial appendage, AML: anterior mitral leaflet, PML: posterior mitral leaflet.

**Figure 17:**
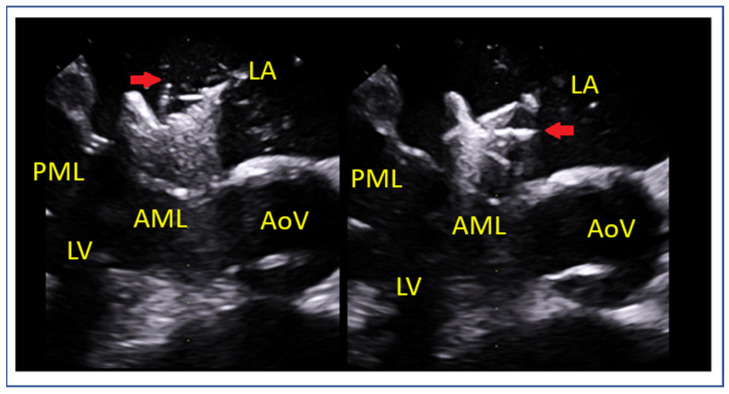
Testing the grippers (indicated by red arrow) to ensure proper function. LA: left atrium, LV: left ventricle, AoV: aortic valve, AML: anterior mitral leaflet, PML: posterior mitral leaflet.

**Figure 18:**
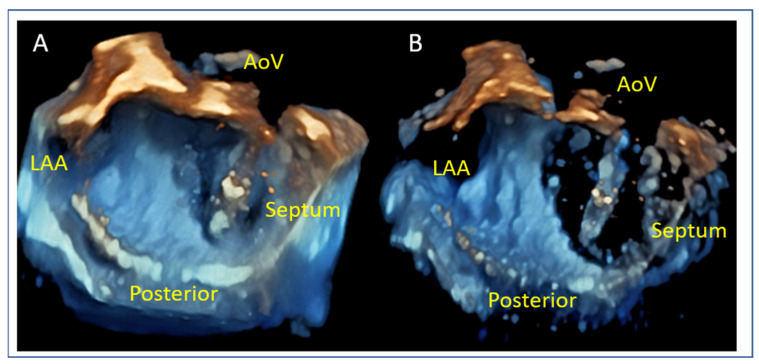
Three-dimensional mitral valve shows device orientation. (**A**) Perpendicularity control after crossing mitral valve to check optimal orientation. (**B**) After the device is advanced into the left ventricle (LV), its orientation can be assessed using 3D echocardiography. Reducing the gain during imaging enhances the clarity of the clip and its position, allowing for more precise visualization of its alignment in relation to the coaptation line. AoV: aortic valve, LAA: left atrial appendage.

Visualizing both device arms in the long-axis view (mid-esophageal 135°) is not a definitive confirmation but can serve as a rough predictor of good alignment. Device alignment is more accurately assessed using 3D imaging, which provides a clear perspective of the device’s orientation in relation to the coaptation line. Proper alignment should be perpendicular to the coaptation line (Figure 16). Before advancing the device into the left ventricle (LV), it is essential to test the functionality of the grippers to ensure they are working properly and to understand which knob operates each gripper (Figure 17). Device perpendicularity is reevaluated after crossing mitral valve, as seen in Figure 18.

### 9.4. Leaflet Grasping

**Figure 19:**
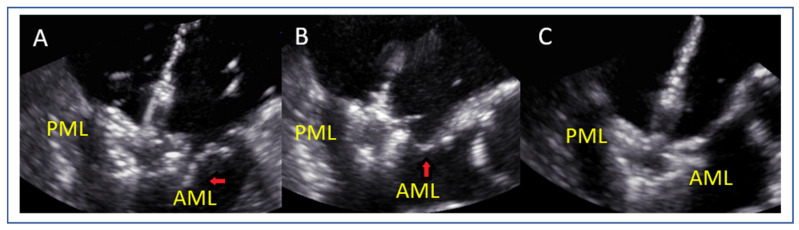
(**A**) Failure to grasp anterior leaflet (indicated by red arrow), (**B**) partial capture of anterior leaflet (red arrow), (**C**) complete capture for both leaflets. AML: anterior mitral leaflet, PML: posterior mitral leaflet.

Inadequate leaflet grasping can result in leaflet detachment, posing significant challenges. During M-TEER procedures, simultaneous grasping is the standard method used to capture both leaflets concurrently. If the initial capture is suboptimal, leaflet optimization is performed by reopening the gripper on the affected side to recapture the compromised leaflet. It is crucial to maintain the clip’s orientation during these adjustments, as even minor twisting can create excessive tension and increase the risk of leaflet injury or tear. Although independent grasping, where each leaflet is captured individually, is available, it is rarely used due to the risk of clip twisting and the associated complications [39]. Precise adjustments are critical to achieving optimal outcomes while minimizing the likelihood of complications (Figure 19).

### 9.5. Device Deployment and Release

**Figure 20:**
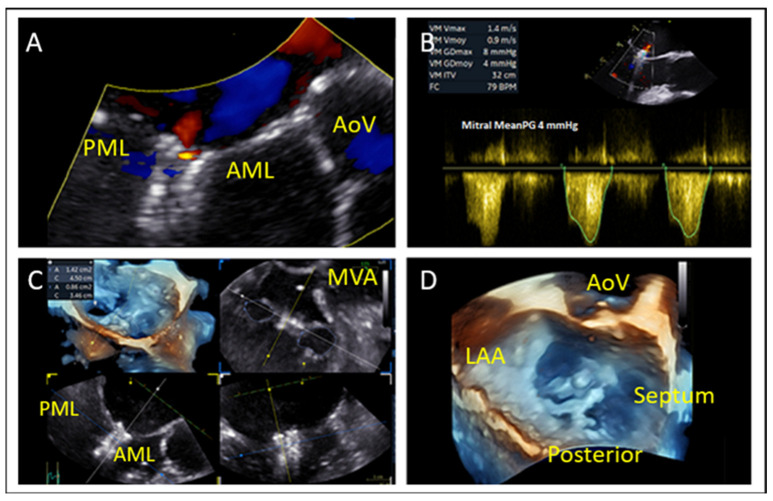
Post-device deployment echocardiographic assessment. (**A**) Color Doppler imaging reveals trace residual mitral regurgitation, indicating minimal leakage after device deployment. (**B**) Continuous-wave Doppler evaluation demonstrates a mean trans-mitral gradient of <5 mmHg, indicating the absence of significant iatrogenic mitral stenosis. (**C**) Three-dimensional multi-planar reconstruction confirms an adequate mitral valve area (MVA ≥ 1.5 cm^2^). (**D**) Three-dimensional image after device implantation demonstrates good tissue bridging. AoV: aortic valve, LAA: left atrial appendage, AML: anterior mitral leaflet, PML: posterior mitral leaflet, MVA: mitral valve area.

Prior to releasing the device, it is critical to assess both the severity and location of any residual mitral regurgitation (MR). Identifying whether the residual jet is medial or lateral helps to understand if an additional device is needed and where it should be implanted. Equally important is confirming that the mitral mean gradient remains within acceptable limits, as significant iatrogenic mitral stenosis might occur. This is generally defined by a mitral valve area (MVA) ≥ 1.5 cm^2^ and a mean trans-mitral gradient < 5 mmHg (Figure 20) [48].

**Figure 21:**
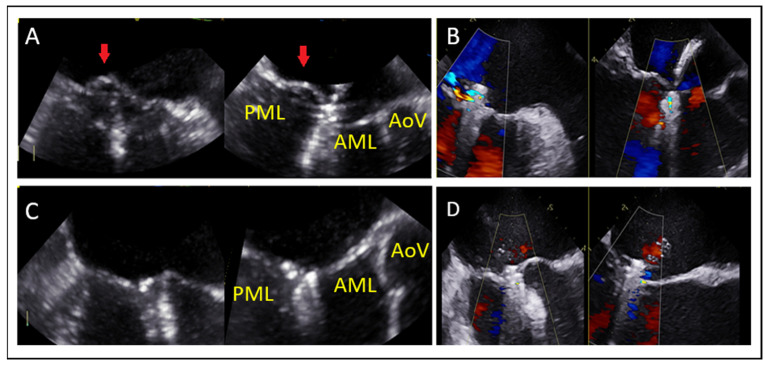
Residual leaflet prolapse after the deployment of the first device and subsequent correction with an additional device implanted medially to the first. (**A**) A 2D echocardiographic view demonstrates persistent leaflet prolapse, medially to the initial device. (**B**) The corresponding color Doppler image reveals a regurgitant jet at the medial aspect, indicating residual mild MR. (**C**) A 2D echocardiographic view, similar to (**A**), shows no residual prolapse after the placement of a second device, confirming optimal anatomical correction. (**D**) The corresponding color Doppler image, similar to (**B**), demonstrates non-significant residual MR. AML: anterior mitral leaflet, PML: posterior mitral leaflet, AoV: aortic valve.

In degenerative MR (DMR), special attention should be given to the underlying leaflet pathology, such as flail or prolapse. Regardless of the residual MR severity, correcting the primary anatomical defect is essential in order to stabilize the valve structure and minimize the risk of future device detachment. Thus, device placement should be guided by anatomical leaflet correction, rather than residual jet severity alone (Figure 21).

Following device deployment, a successful outcome is typically defined by a reduction in MR severity to trace or mild levels. According to the Mitral Valve Academic Research Consortium (MVARC) criteria, an optimal result corresponds to post-procedural MR of none or trace, while an acceptable result involves at least a one-grade reduction from baseline MR severity (e.g., from severe to moderate) [48]. Doppler measurements, including mitral gradient and valve area, should be interpreted within the context of the patient’s hemodynamic status, as systemic hypotension can underestimate MR severity by reducing driving force across the regurgitant orifice and suppressing the color Doppler jet appearance.

Additional echocardiographic findings provide further confirmation of successful MR reduction. The resolution of systolic flow reversal in the pulmonary veins suggests effective decompression of left atrial pressure [49], while the presence of spontaneous echo contrast (SEC) in the left atrium following device placement indicates blood flow stagnation and is an indirect marker of significant MR reduction [50].

Before completing the procedure, device stability must be carefully evaluated. In cases of rocking or malposition, additional device implantation may be necessary [51], provided that mitral valve area, trans-mitral gradient, and leaflet quality remain suitable for further intervention. A stable device not only ensures immediate procedural success but also mitigates the risk of complications such as subacute device detachment [51].

## 10. Conclusions

Mitral transcatheter edge-to-edge repair (M-TEER) has significantly advanced the management of mitral regurgitation (MR), combining theoretical insights with clinical practice. Its success depends on both meticulous procedural execution and appropriate patient selection. Echocardiography plays a central role by providing detailed assessment of mitral valve anatomy and ventricular remodeling, ensuring optimal results. By integrating imaging, patient characteristics, and technique, M-TEER transforms complex valve disease into a treatable condition, offering lasting improvement in patient care.

## Figures and Tables

**Table 1:** Clinical criteria for optimal patient selection for M-TEER.

COAPT-Eligible Characteristics	COAPT-Ineligible Characteristics
Ischemic or non-ischemic cardiomyopathy with LVEF = 20–50% LV end-systolic diameter ≤ 70 mm Moderate-to-severe (≥3+) or severe (4+) secondary MR Symptomatic heart failure (NYHA Class II, III, or ambulatory IV) despite optimal medical therapy and cardiac resynchronization therapy At least one hospitalization for HF the previous year or elevated natriuretic peptides MV anatomy suitable for M-TEER	Severe disability/frailty Hypertrophic cardiomyopathy, restrictive cardiomyopathy, constrictive pericarditis Infiltrative cardiomyopathies (amyloidosis, haemochromatosis, sarcoidosis) Estimated sPAP > 70 mmHg Moderate or severe right ventricular dysfunction Hemodynamic instability or cardiogenic shock Mitral valve orifice < 4.0 cm^2^ by site-assessed TTE Coronary, aortic or tricuspid valve disease requiring surgery

**Table 2:** Anatomical selection criteria for M-TEER.

Optimal	Challenging	Unsuitable
A2/P2 pathology	Commissural (A1/P1, A3/P3)	MVA < 3.0 cm^2^
MVA > 4 cm^2^	MVA > 3.0 cm^2^	Posterior leaflet length <7 mm and cleft
Posterior leaflet lenght > 10 mm	Posterior leaflet length 7–10 mm or cleft	Calcification in grasping zone
No calcification	No calcification in grasping zone, annulus calcification	Rheumatic mitral valve disease
DMR criteria: flail gap < 10 mm, flail width < 15 mm	DMR criteria: flail width > 15 mm	Multiple segments, Barlow
FMR criteria: tenting height < 10 mm	FMR criteria: Tenting height > 10 mm	
Normal leaflets and mobility	Carpentier IIIB	

## Data Availability

All data supporting this article are available upon reasonable request.

## Abbreviations

The following abbreviations are used in this manuscript:

AFMR	Atrial functional MR
BTT	Bridge-to-transplant
CRT	Cardiac resynchronization therapy
DMR	Degenerative mitral regurgitation
EROA	Effective regurgitant orifice area
GDMT	Guideline-directed medical therapy
HF	Heart failure
IVC	Inferior vena cava
LA	Left atrium
LAVI	Left atrial volume index
LV	Left ventricle
LVAD	Left ventricular assist device
LVEDV	Left ventricular end-diastolic volume
LVEF	Left ventricular ejection fraction
LVESD	Left ventricular end-systolic diameter
MPR	Multiplanar reconstruction
MR	Mitral regurgitation
M-TEER	Mitral transcatheter edge-to-edge repair
MV	Mitral valve
MVA	Mitral valve area
NYHA	New York Heart Association Class
PCI	Percutaneous coronary intervention
PMR	Primary mitral regurgitation
RF	Regurgitant fraction
RVol	Regurgitant volume
SMR	Secondary mitral regurgitation
SPAP	Systolic pulmonary artery pressure
TAVI	Transcatheter aortic valve implantation
TEE/TOE	Transesophageal echocardiography
TR	Tricuspid regurgitation
VC	Vena contracta
